# Metabolic behavior for a mutant *Oenococcus oeni* strain with high resistance to ethanol to survive under oenological multi-stress conditions

**DOI:** 10.3389/fmicb.2023.1100501

**Published:** 2023-03-09

**Authors:** Ángela Contreras, Gabriela Díaz, Sebastián N. Mendoza, Mauricio Canto, Eduardo Agosín

**Affiliations:** ^1^Applied Microbiology Laboratory, Center for Biotechnology of Natural Resources, Faculty of Agricultural and Forestry Sciences, School of Biotechnology, Universidad Católica del Maule, Talca, Chile; ^2^Laboratory of Biotechnology, Department of Chemical and Bioprocess Engineering, School of Engineering, Pontificia Universidad Católica de Chile, Santiago, Chile; ^3^Systems Biology Lab, Amsterdam Institute of Molecular and Life Sciences (AIMMS), Vrije Universiteit Amsterdam, Amsterdam, Netherlands

**Keywords:** *Oenococcus oeni*, metabolism, malolactic fermentation, random mutagenesis, ethanol resistance

## Abstract

Malolactic fermentation (MLF) positively influences the quality of the wine, and it occurs as a result of a lactic acid bacteria’s metabolism, mainly of the *Oenococcus oeni* species. However, delays and halting of MLF are frequent problems in the wine industry. This is mainly because *O. oeni*’s development is inhibited by different kinds of stress. Even though the sequencing of the genome of the PSU-1 strain of *O. oeni*, as well as other strains, has made it possible to identify genes involved in the resistance to some types of stress, all of the factors that could be involved are still unknown. With the aim of contributing to this knowledge, the random mutagenesis technique was used in this study as a strategy for genetic improvement of strains of the *O. oeni* species. The technique proved to be capable of generating a different and improved strain when compared to the PSU-1 strain (the parent from which it descends). Then, we evaluated the metabolic behavior of both strains in three different wines. We used synthetic MaxOeno wine (pH 3.5; 15% v/v ethanol), red wine (Cabernet Sauvignon), and white wine (Chardonnay). Furthermore, we compared the transcriptome of both strains, grown in MaxOeno synthetic wine. The specific growth rate of the E1 strain was on average 39% higher in comparison to the PSU-1 strain. Interestingly, E1 strain showed an overexpression of the OEOE_1794 gene, which encodes a UspA-like protein, which has been described as promoting growth. We observed that the E1 strain was able to convert, on average, 34% more malic acid into lactate than the PSU-1 strain, regardless of the wine being used. On the other hand, the E1 strain showed a flux rate of fructose-6-phosphate production that was 86% higher than the mannitol production rate, and the internal flux rates increase in the direction of pyruvate production. This coincides with the higher number of OEOE_1708 gene transcripts observed in the E1 strain grown in MaxOeno. This gene encodes for an enzyme fructokinase (EC 2.7.1.4) involved in the transformation of fructose to fructose-6-phosphate.

## Introduction

Lactic acid bacteria (LAB) are microorganisms used in the wine industry to manufacture a wide variety of products. An important and valuable feature is that these bacteria can deacidify their growth media; for this reason, they are regularly used in fermented product production (Derkx et al., 2014).

Malolactic fermentation (MLF) positively influences the quality of wine, contributing to its microbiological stability and increasing the complexity of its flavors (Davis et al., 1985; Henschke, 1993; Bartowsky et al., 2002). MLF occurs as a result of lactic acid bacteria’s metabolism and consists of the conversion of L–(+)–malate to L–(+)–lactate and CO_2_, by the action of the malolactic enzyme (MLE) (Caspritz and Radler, 1983). Both, the consumption of L-malate and the duration of MLF, depend on the biomass of lactic acid bacteria and the activity of its malolactic enzyme, which is influenced by the adaptation capacity of bacteria (Guzzo and Desroche, 2009).

*Oenococcus oeni* strains are recognized for their high tolerance to stressful conditions present in the winemaking process, such as, low pH, the presence of SO_2_ and ethanol; and therefore, these strains are the main ones responsible for MLF (Tracey and Britz, 1987; Van Vuuren and Dicks, 1993; Versari et al., 1999).

Despite the metabolic and adaptive capacities of *O. oeni*, delays and halting MLF are frequent problems in the wine industry (Eglinton and Henschke, 1996; Gockowiak and Henschke, 2003; Malherbe et al., 2007). These problems are mainly from the development of *O. oeni* being inhibited by compounds present in the culture medium, such as ethanol and sulfur dioxide (SO_2_) (Britz and Tracey, 1990; Patynowski et al., 2002; Gockowiak and Henschke, 2003). Consequently, numerous selection studies have been focused on isolating *O. oeni* strains which are resistant to these inhibitors (Malacrinò et al., 2003; Izquierdo et al., 2004; US 8114449, Guzzo and Desroche, 2009; Solieri and Giudici, 2010; Marques et al., 2011). Among the main strategies, for a natural selection of a microorganism starter culture, are adaptive evolution (directed evolution), random mutagenesis (UV or Chemical induced), and phenotypic analysis of natural isolates (Akcil, 2004; Li et al., 2015). Some studies have generated an evolved *O. oeni* using: adaptive evolution (Betteridge et al., 2018; Jiang et al., 2018) and UV-induced random mutagenesis (Li et al., 2015). Chemical mutagenesis has not been used in *O. oeni* but it has been used in other lactic acid bacteria such as *Lactobacillus bulgaricus* and *Streptococcus thermophiles* (Ibrahim and O'Sullivan, 2000). However, these studies have not performed a comparative transcriptomic, metabolic, or genomic analysis between the improved strains and their parent strain. The comparison between individuals that widely share a genotype and those that differ in a certain phenotype generates a great opportunity to identify those changes responsible for that differentiation.

Our current research is performed on the context of continuous research on the topic. Our study group built the first genome-scale metabolic model of *O. oeni* (Mendoza et al., 2017). Subsequently, we carried out an analysis of the effects of different concentrations of ethanol on the distribution of metabolic fluxes of bacteria. This study provided a global vision of how the increase in ethanol content exerts a differentiated physiological response in *O. oeni* (Contreras et al., 2018). Likewise, new studies have emerged that analyze the transcriptomic and metabolic changes by an *O. oeni* strain cultured under acid stress conditions, characterizing its behavior (Onetto et al., 2021; Qi et al., 2021).

Even though sequencing of PSU-1 genome, as well as other strains of this species, has made it possible to identify genes involved in resistance to various types of stress (Mills et al., 2005; Borneman et al., 2012), the generation of a fitter strain is often a combination of multiple mutations in the genome. Thus, it is very challenging to predict changes that will results in an improved strain. In the present study, random chemical mutagenesis strategy allowed us to obtain a strain E1 that was resistant to ethanol stress, which was tested in synthetic and natural wines. By performing a transcriptomics analysis on both strains, we were able to identify changes in the transcription levels of genes related to stress resistance, and also predict how metabolic fluxes were redistributed using metabolic models. These results contribute to identify key genes related ethanol resistance to understand how these changes influence *O. oeni* metabolism.

## Materials and methods

### Strains and growth conditions

An *Oenococcus oeni* PSU-1 ATCC™ (Garvie, 1967) preculture was prepared from a frozen stock by inoculating 100-ml Erlenmeyer flasks containing an FT80 medium, at a pH of 4.8 (Guilloux-Benatier et al., 1995), and incubated at 28°C. In our previous studies, we observed that this pH allowed for the optimization of *O. oeni*’s growth under ethanol stress conditions. For this reason, we used this pH in the selection step (Contreras et al., 2018). FT80 was used because it imitates wine composition and allows us to evaluate the malolactic fermentation process. Growth was followed by measuring optical density at 600 nm, using a Spectronic 20™ spectrophotometer and an Infinite 200 PRO Microplate reader (Tecan, Switzerland). PSU-1 was called “parental strain” and the mutant strain obtained during this study was called “E1” and/or “mutant strain.”

### Evaluation of initial strain resistance to ethanol and potassium metabisulfite

*Oenococcus oeni* PSU-1 strain was grown in 10 mL of FT80 medium with a pH of 4.8, adding ethanol as a stressor. An ethanol concentration of 10, 15, or 20%v/v was used in the culture medium. Similarly, the strain was evaluated by adding potassium metabisulfite as a stressor at 50, 100, or 200 mg/L in the culture media, which was prepared in the moment. Every culture was inoculated with approximately 3.2 × 10^8^ UFC/mL cells to achieve an optical density of 0.2 in the flask. Cultures were sampled periodically, for 9 days. We considered growth when the cultures showed an optical density equal to or higher to 0.3.

### Ethyl methane sulfonate mutagenesis procedure

The PSU-1 strain was grown at 28°C in 70 mL of FT80 medium to obtain an optical density of 0.2 (3.2 × 10^8^ UFC/mL). The cultures were centrifuged at 3000 rpm for 5 min, and cells were washed and suspended in 10 mL of 50 mM H_2_HPO_4_/K_2_HPO_4_ buffer (pH 7.5). Suspended cells were mixed with ethyl methane sulfonate (EMS) and incubated at 30°C, with a gentle agitation for 30 min. The PSU-1 cells were treated individually using 5% v/v of EMS, this addition corresponded to a 99.5% of lethality. At the end of the incubation process, the cell was treated using buffer sodium thiosulfate 10% w/v for 5 min. Later, they were centrifuged at 3,000 rpm for 3 min, the supernatant was eliminated, and cells were washed two times using buffer and distilled water. Finally, the cells were suspended in distilled water, and then 100 μL of suspension were plated on FT80 agar medium and incubated at 28°C for 24 h. Subsequently, around 10 colonies were taken per plate and were evaluated with a stressor using FT80.

#### Mutant selection

Colonies obtained in soft agar plates were grown individually in 10 mL of FT80 broth, and later were inoculated in 10 mL of FT80 with the concentration selected from the stressor. PSU-1 strain was evaluated using 15%v/v of ethanol and 100 mg/L of potassium metabisulfite. Every culture was incubated at 25°C, without stirring, and were sampled periodically for 10 days. We considered growth when the cultures showed an optical density equal or higher to 0.3 (Supplementary Table S1, Supplementary Figures S1, S2, Supplementary material). Finally, the obtained mutant strains were stored at −80°C as glycerol stocks, using glycerol at 50% v/v.

Subsequently, mutant cells were inoculated in 100 mL flasks containing 75 mL of FT80 at pH 4.8, in duplicate. The medium was inoculated at an optical density of 0.2 (at 600 nm), and the cultures were incubated at 25°C, without stirring. The growth was monitored periodically for 10 days (Supplementary Table S1, Supplementary Figures S1, S2, Supplementary material). At the end of this stage, only one strain was able to grow, in duplicate, and therefore it was selected and stored at −80°C as glycerol stock, using glycerol at 50% v/v. The mutant strain was initially called E1.

### Evaluation of the growth and malolactic activity of selected *Oenococcus oeni* strains using synthetic wine using different pH

The mutant strain selected (E1) was evaluated using synthetic wine called MaxOeno, with 15% v/v of ethanol, which was previously developed by our group (Contreras et al., 2018). MaxOeno is a fully defined culture medium, which enables the quantification of substrate consumption and metabolite production; pH was adjusted at 3.5 or 4.8 using KCl and HCl.

Mutant strain E1 was inoculated into 100 mL flasks containing 75 mL of MaxOeno at pH 3.5 or 4.8, in duplicate. The medium was inoculated at an optical density of 0.2 (at 600 nm) and the cultures were incubated at 25 ° C, without shaking. Samples were taken periodically for 10 days, determining growth (by optical density), malate consumption, and lactate production (by HPLC).

### Evaluation of the growth and malolactic activity of selected *Oenococcus oeni* strain in natural wine (*Cabernet Sauvignon and Chardonnay*)

The red and white grape musts used were provided by the Cono Sur™ vineyard and obtained from the city of Chimbarongo, VI Region of Chile. Both were characterized in terms of pH, assimilable nitrogen, glucose, fructose, total SO_2_, and free SO_2_ obtained for each variety. The composition of the Chardonnay must was: glucose 100 g L^−1^, fructose 100 g L^−1^, pH 3.2; assimilable nitrogen 415 mg N L^−1^. The composition of the Cabernet Sauvignon must was: glucose 100 g L^−1^, fructose 100 g L^−1^, pH 3.5, assimilable nitrogen 300 mg N L^−1^.

Finally, the red and white grape musts showed 32 and 28 mg L^−1^ of free SO_2_, and also, 88 and 90 mg L^−1^ of total SO₂, respectively. The total acidity was determined by alkalinity with a bromothymol blue indicator, obtaining 1.617 and 3.185 g L^−1^ of total acidity (considering sulfuric acid) for red and white must, respectively. In addition, we determined in those musts a total acidity of 2.475 and 4.875 g L^−1^ (considered as tartaric acid), respectively. The total acidity was determined by both methods, as described in the technical instructions for grape musts analysis (Resolución N° 8.542, 2013).

The wines were produced using EC1118 ™ (Lallemand) yeast, which was inoculated at a concentration of 1×10^6^ cells/mL in 700 mL of grape must, using 1-liter reactors.

The Chardonnay wine composition was glucose 2.3 g L^−1^; fructose 2.3 g L^−1^; pH 3.2; assimilable nitrogen 200 mg N L^−1^, and 14% v/v of ethanol. The Cabernet Sauvignon wine composition was glucose 1.9 g L^−1^, fructose 7.2 g L^−1^, pH 3.5; assimilable nitrogen 150 mg N L^−1^, and ethanol 15.5% v/v. In both cases, the alcoholic fermentation was concluded when less than 10 g L^−1^ of residual sugar was detected.

Subsequently, malolactic fermentation was carried out, inoculating the E1 mutant strain or the PSU-1 strain in each wine produced. The parental strain was subjected to adaptation to ethanol and pH because it was not able to grow without this adaptation. For this, they were cultivated in MaxOeno defined synthetic wine in which the pH varied from 4.8 to 3.5, and the ethanol from 0 to 12%. The mutant strain E1 was inoculated directly. Both strains were inoculated at a 0.2 optic density (3.2×10^8^ cells/ml), in duplicate.

Samples were taken periodically to monitor their growth; the supernatants were frozen to determine the consumption and production of compounds.

### Characterization of grape musts and wine

Free and total SO₂ were determined by the Ripper’s method, as described by Schwarze and Edmundo (2000). Moreover, total acidity was obtained by titration, and 10 mL of grape must was placed in a 100 mL beaker, 3 drops of bromothymol blue indicator were added. Titration began with 0.1 N NaOH with constant stirring until the green indicator turned to a blue-green color.

On the other hand, pH was determined using a pH meter HI2221™ (Hanna instruments, United States). The presence of reducing sugars in the grape must was determined through a refractometer and a high-performance liquid chromatography (HPLC) with the LaChrom L-7000 HPLC system (Hitachi, Japan). The presence of reducing sugars in the wine was determined using only HPLC.

Finally, nitrogen concentration was determined by UHPLC–MS, using the Ultimate 3,000 system (ThermoScientific, United States). The compounds were separated through the RP-18 ion exchange column (LiChrospher®, Germany) as described by Contreras et al., 2018.

### Chromatographic analyses

Samples obtained from each culture were centrifuged and the supernatant was collected, and an aliquot was injected in a Lachrom L-700 HPLC system (Hitachi, Japan) equipped with a Diode Array and a Refractive Index detector (Merck Hitachi, Japan). Organic acids, alcohols and sugars were separated using an Aminex HPX-87H ion exchange carbohydrate-organic acid column (Bio-Rad, United States), and quantified as described previously (Varela et al., 2003).

### Transcriptomic analysis using Illumina hi-seq2500

The gene expression profiles of the mutant strain and its parent were obtained using RNAseq. Massive transcriptome sequencing was performed using the HiSeq 2,500® technique, where 10 million reads per gene were considered for mapping. The transcriptome sequencing with ribosomal depletion, and the data analysis services were hired at the Molecular Research DNA LAB (Texas, United States).

Bacterial RNA was extracted using kit E.Z.N.A™ (Omega Biotek, United States) and treated with DNAsa I (Omega Biotek, United States), following the manufacturer’s instructions. All the samples were suspended on DEPC water and dried using RNAstable® tubes (Biomatrica, United States).

The concentration of total RNA was determined using the Qubit® RNA Assay Kit (Life Technologies); it was around 200 ng/μL of RNA in each case. RNA integrity value (RIN) was determined by using the Agilent RNA 6000 Nano Reagents and the RNA Nano Chips in Agilent 2,100 Bioanalyzer (Agilent Technologies), obtaining a RIN value of about 2. To remove DNA contamination 0.5–1.5 ug of total RNA was cleaned using Baseline-ZERO™ DNase (Epicentre) following the manufacturer’s instructions, followed by purification using the RNA Clean & Concentrator-5 columns (Zymo Research). DNA free of RNA samples were used for rRNA removal by using Ribo-Zero™ Magnetic Gold Kit (Epidemiology; Illumina). Final purification was performed using the RNA Clean & Concentrator-5 columns (Zymo Research). rRNA depleted samples were used for library preparation using the TruSeq™ RNA LT Sample Preparation Kit (Illumina) according to the manufacturer’s instructions. Following the library preparation, the final concentration of all the libraries were measured by using the Qubit® dsDNA HS Assay Kit (Life Technologies), and the average library size was determined using the Agilent 2,100 Bioanalyzer (Agilent Technologies), which was approximately 33 ng/μL. The libraries were then pooled in equimolar ratios of 2 nM, and 8pM of the library pool was clustered using the cBot (Illumina), and paired-end sequenced for 300 cycles using the HiSeq 2,500 system (Illumina).

Raw reads were normalized through RPKM (reads per kilo base per million reads) method using the Qseq program (Conesa et al., 2016). Publicly available transcriptome data sets were downloaded from the NCBI’s database and they were mapped using the QSeq program of DNASTAR. Fold change for each gene was calculated as:


(S1) $$\mathrm{Fold}\;\mathrm{Change}=\log_{2}\;\frac{\mathrm{RPKM}\;\left(E1\right)}{\mathrm{RPKM}\left(PSU1\right)}$$

All the information is in the NCBI database, GEO accession GSE217343 and token mberokakzfirnyl.^1^

### Gene expression validation using QPCR

For cDNA preparation, 5 μg of RNA was incubated with 1 unit of DNase I at 37°C for 30 min. Afterwards, the instructions of the manufacturer for M-MLV were followed. The cDNA obtained was used as template in the real time PCR reaction (QPCR). The QPCR reaction was carried out in a final volume of 10 μL. The reaction mixture contained 5 μL of Brilliant II SYBR Green QPCR Master mix (Stratagene, United States), 0.2 μM of each primer (Supplementary Table S2; Supplemental material), and 10 ng/μL of cDNA. The QPCR reaction was carried out in an AriaMx Real-Time PCR equipment (Agilent Technologies, United States) under the following conditions: 50°C for 2 min, 95°C for 1 min, 45 cycles of 95°C for 3 s, 60°C for 10s, and 72°C for 20s, a melting analysis at 95°C for 30s, at 60°C for 30 s, and at 95°C for 30s. Quantification of relative gene expression was done by using the mathematic method (Livak and Schmittgen, 2001) and normalized with *OEOE_005* gene. This gene codes for a dehydrogenase-like protein, and it was selected as a housekeeping gene because each time it was evaluated it showed no changes in its expression between the strains.

### Statistical analysis

Differences between measurements were determined using the Mood test (Gibbons and Chakraborti, 2011) with the Statgraphics Centurion XIX statistical software. We considered differences significant when *p*-values were less than 0.05.

### Data integration using the genomic scale metabolic iSM454 model

#### Flux balance analysis

Flux balance analysis was carried out through the iSM454 model, developed by Mendoza et al. (2017), to analyze the highest growth phase of the PSU-1 and E1 strain in the different culture media used. In order to do so, the following fluxes (mmol gDCW^−1^ h^−1^) were set with experimental values: the substrates’ glucose, fructose, citrate, malate, and the mannitol, erythritol, L-lactate, and acetate products. In addition, experimental biomass and NGAM estimated in this work were fixed. For the visualization of metabolic pathways, the ESCHER® web platform was used.

All the information is in the Git-Hub platform.^2^ Token https://github.com/Maucicio/Strain-analysis-main-Public-.

#### Prediction of non-growth associated

Non-growth associated maintenance (NGAM) was estimated by setting the specific production and consumption rates of the experimentally measured compounds, as described above for FBA. Thereby, flux through the NGAM reaction was progressively increased from 0 to 10 mmol gDCW^−1^ h ^−1^. For each cycle, biomass production rate was maximized, and the prediction error was assessed comparing biomass prediction *in silico* to experimental biomass. The NGAM flux that allowed the lowest biomass prediction error was selected.

## Results

**Figure 1:**
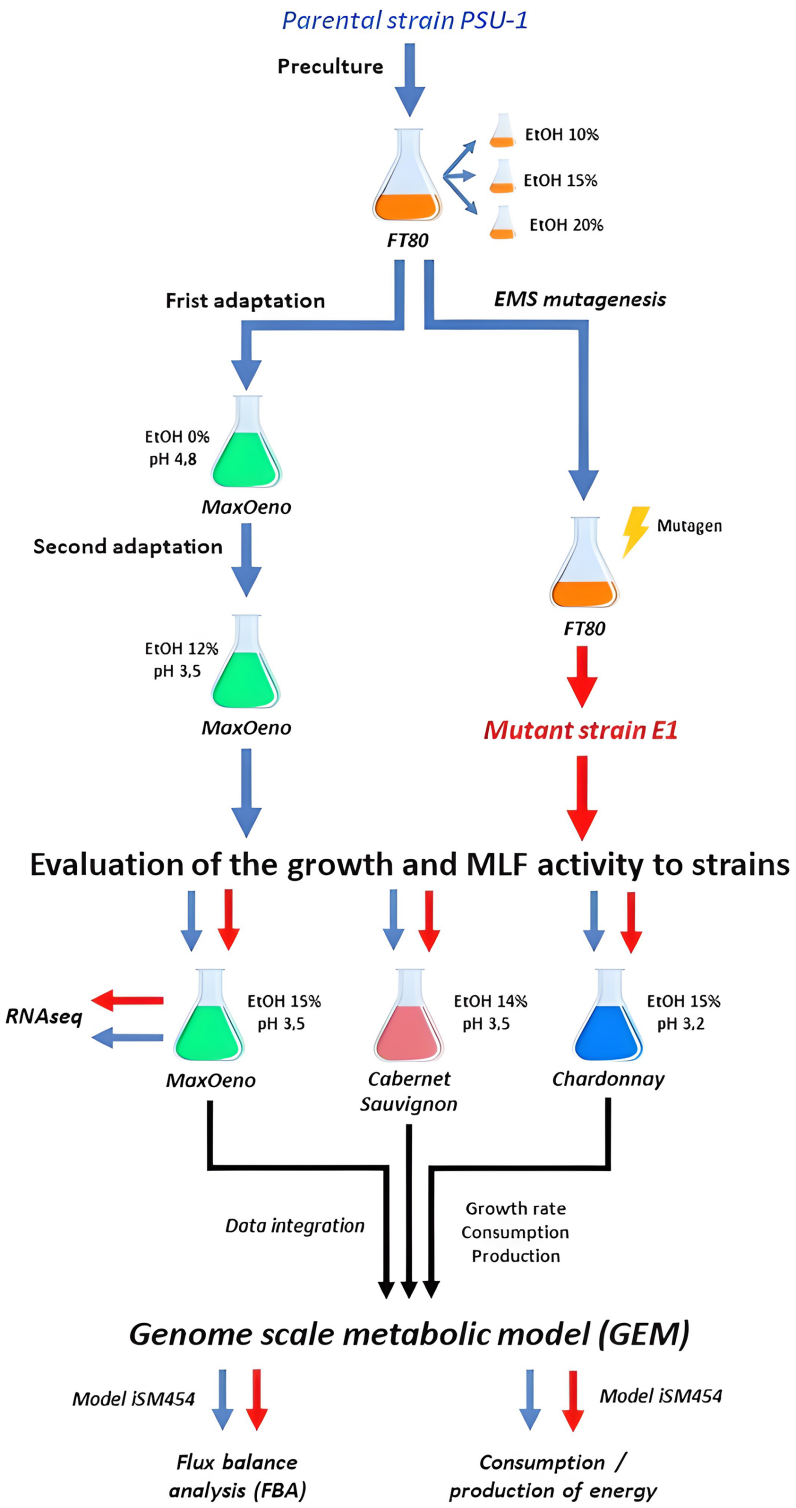
Full experimental process scheme.

In this study, an *Oenococcus oeni* ethanol-resistant strain was obtained by random mutagenesis. This strain and its parent strain were compared at the transcriptomic (using a synthetic wine) and metabolic levels. The metabolism was studied using synthetic wine (MaxOeno), Cabernet Sauvignon, and Chardonnay (Figure 1).

### Evaluation of the mutant strain E1 and its parental strain PSU-1, using MaxOeno (defined synthetic wine) at pH 4.8, as the last selection step

The mutant strain E1 was able to grow in 15% ethanol and a high malic acid concentration, as stress factors, showing a higher biomass formation compared to the PSU-1 strain, under the same culture conditions (Supplementary Figure S3A, Supplementary material). Furthermore, E1 had a higher consumption of malate and, therefore, a higher production of lactate (Supplementary Figures S3B,C, Supplementary material). Besides, E1 showed a higher consumption of glucose and fructose (Supplementary Figures S3D,E, Supplementary material).

### Effect of wine type on the growth of *Oenococcus oeni* PSU-1 and E1 strain

**Figure 2:**
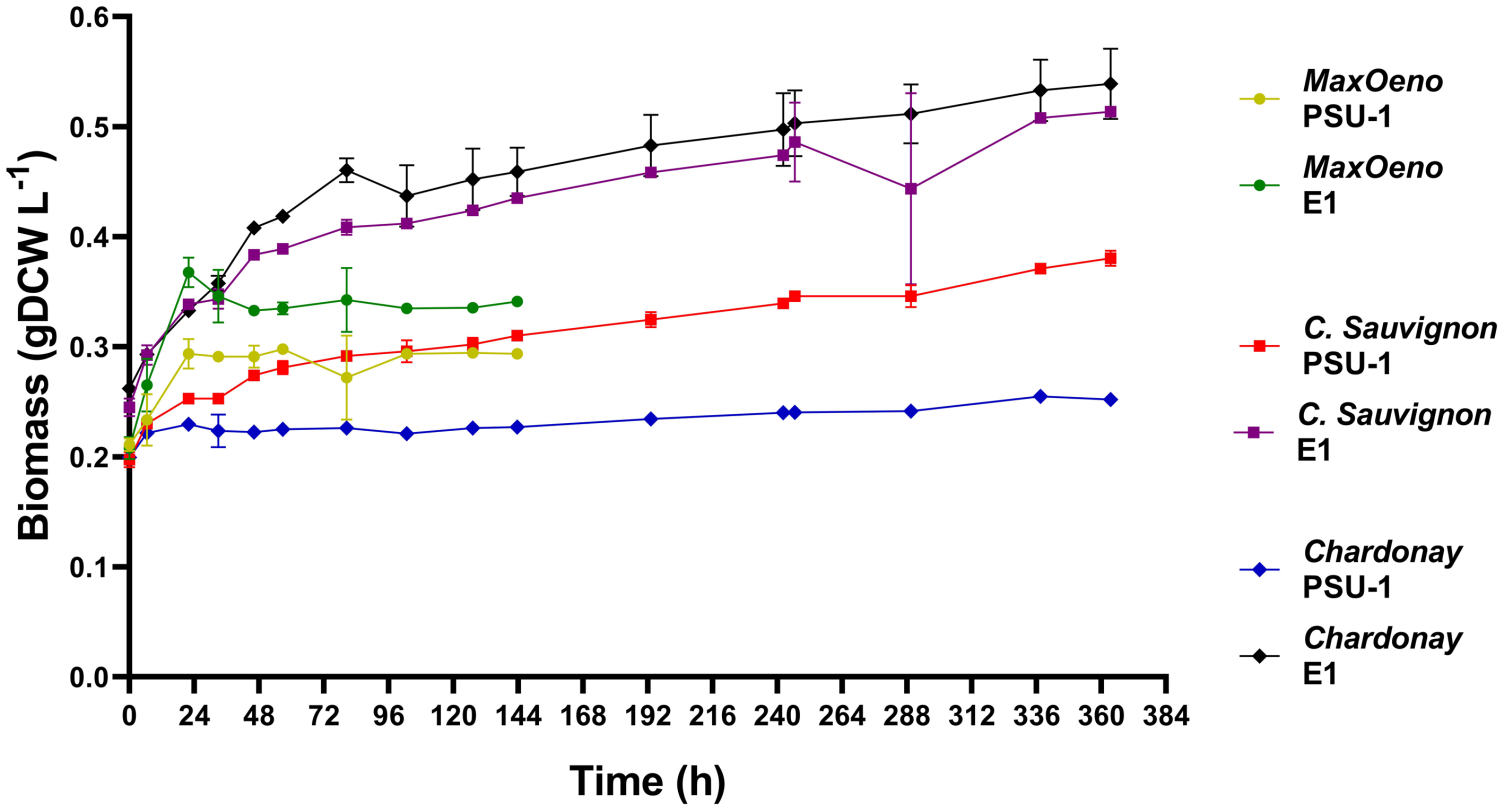
Evaluation of the growth of the PSU-1 and the E1 strains in different types of wine.

We evaluated the growth of the PSU-1 and E1 strains on three wine types (Figure 2). When comparing the growth capacity of the strains, we observed that the E1 strain is better adapted to the stress conditions of each wine and grows more than the PSU-1 strain. The E1 strain was able to complete the exponential phase earlier in synthetic wine (22 h) than in natural white and red wine (80 h). However, the highest concentration of biomass produced was observed in both natural wines at the 80 h mark. It was observed that E1 had a higher biomass production in Cabernet Sauvignon (48% more) and Chardonnay (46% more) compared to biomass production in MaxOeno (Table 1). Likewise, the PSU-1 strain showed higher biomass production in Cabernet Sauvignon (44% more) than in MaxOeno, however, it produced lower biomass in Chardonnay (62% less). Among the evaluated wines, white wine (Chardonnay) had a higher impact on the growth of the PSU-1 strain. In general, it was observed that E1 had a higher biomass production, in Cabernet Sauvignon (67% more), MaxOeno (61% more) and Chardonnay (18% more), compared to PSU-1 (Table 1).

**Table 1:** Total consumption and production of compounds of strains PSU1 and E1 cultivated in different wines (*) (φ).

Wine	Biomass production (gDCW L^−1^)		Glucose consumption (g L^−1^)		Fructose consumption (g L^−1^)		Mannitol production (g L^−1^)		Erythritol production (g L^−1^)	
	PSU-1	E1	PSU-1	E1	PSU-1	E1	PSU-1	E1	PSU-1	E1
MaxOeno pH 3.5	0.08^Aa^ ± 0.00	0.13^Ab^ ± 0.01	3.05^Aa^ ± 0.33	3.05^Aa^ ± 0.05	1.25^Aa^ ± 0.86	2.16^Ab^ ± 0.11	0.33^Aa^ ± 0.02	0.33^Aa^ ± 0.09	0.23^Aa^ ± 0.02	0.38^Ab^ ± 0.00
Cabernet Sauvignon	0.18^Ba^ ± 0.00	0.27^Bb^ ± 0.01	0.81^Ba^ ± 0.29	0.83^Ba^ ± 0.38	1.88^Ba^ ± 0.23	3.82^Bb^ ± 0.23	ND	0.26^Ab^ ± 0.01	0.32^Ba^ ± 0.05	0.19^Ab^ ± 0.00
Chardonay	0.05^Ca^ ± 0.00	0.28^Ab^ ± 0.03	0.94^Ba^ ± 0.01	1.03^Bb^ ± 0.64	0.51^Ca^ ± 0.11	0.71^Cb^ ± 0.11	ND	ND	0.25^Ca^ ± 0.00	0.25^Ba^ ± 0.04
Wine	Growth rate (¥) (h^−1^)		Malate consumption (g L^−1^)		Lactate production (g L^−1^)		Citrate consumption (g L^−1^)		Acetate production (g L^−1^)	
	PSU-1	E1	PSU-1	E1	PSU-1	E1	PSU-1	E1	PSU-1	E1
MaxOeno pH 3.5	0.01^Aa^ ± 0.0	0.02^Ab^ ± 0.00	0.93^Aa^ ± 0.28	1.37^Ab^ ± 0.30	0.14^Aa^ ± 0.01	0.49^Ab^ ± 0.05	0.07^Aa^ ± 0.01	0.13^Ab^ ± 0.00	4.19^Aa^ ± 0.40	3.45^Ab^ ± 0.00
Cabernet Sauvignon	0.004^Ba^ ± 0.0	0.01^Bb^ ± 0.00	1.42^Ba^ ± 0.24	2.14^Bb^ ± 0.22	0.67^Ba^ ± 0.01	1.90^Bb^ ± 0.03	0.37^Ba^ ± 0.02	1.01^Bb^ ± 0.12	0.13^Ba^ ± 0.06	0.10^Ba^ ± 0.01
Chardonay	0.001^Ba^ ± 0.0	0.01^Bb^ ± 0.00	0.85^Aa^ ± 0.30	1.27^Ab^ ± 0.07	0.91^Ca^ ± 0.16	1.97^Bb^ ± 0.11	0.34^Ba^ ± 0.05	0.35^Ba^ ± 0.03	0.18^Ba^ ± 0.05	0.05^Cb^ ± 0.02

(*) Same letters in the same row or column indicate that there are no statistically significant differences (*p* < 0.05, Mood test) between the reported values. Likewise, capital letters indicate differences or similarities between treatments and lower cases indicate differences or similarities between strains.

¥ The calculations were made considering only the growth phase of the strains in the culture.

(φ) The calculation was made considering total consumption and total production of compound.

The E1 and PSU-1 strain were cultured using MaxOeno 15% v/v of ethanol at pH 3.5 (Supplementary Figures S4, S5, Supplementary material). For this, an adaptation to pH was carried out in PSU-1, as was mentioned above. The cultivated E1 strain showed a higher biomass formation and specific growth rate, compared to the PSU-1 strain (Table 1).

The PSU-1 strain was able to grow and perform MLF in red wine. Moreover, the PSU-1 strain produced a higher biomass in red wine, even more than in synthetic wine (44% more), although with a slower rate of growth (Table 1). Likewise, the E1 strain produced a higher biomass than PSU-1 in red wine (67% more) (Table 1).

### Effect of wine type on metabolites production and substrate consumption of *Oenococcus oeni* PSU-1 and E1 strain

#### MaxOeno (synthetic wine) at pH 3.5

In addition, when we considered the production of metabolites and consumption of nutrients, it was observed that E1 had a higher consumption of L-malate (68% more) and, therefore, a higher production of (D + L)-lactate (29% more) (Table 1). Interestingly, E1 showed a higher total of citrate and fructose consumption than PSU-1 (Table 1), however, total glucose consumption did not show statistically significant differences between the cultures of either strain (Table 1). The higher fructose consumption of E1 was not directly related to mannitol production, and we observed that both strains produced the same concentration for total mannitol. Furthermore, glucose consumption in MaxOeno was higher than fructose consumption, specifically 41 and 71% higher in PSU-1 and in E1, respectively. However, the E1 strain produces more erythritol (60% more) from glucose than PSU-1 (Table 1).

In relation to the total acetate produced, the PSU-1 strain showed a higher production of this metabolite (82% more); compared to E1 (Table 1).

#### Red wine variety Cabernet Sauvignon (pH 3.5)

It was observed that in red wine E1 had a higher consumption of L-malate and, therefore, a higher production of (D + L)-lactate (Table 1). Interestingly, E1 showed a higher total citrate and fructose consumption than PSU-1 (Table 1). However, total glucose consumption did not show statistically significant differences between the cultures of either strain (Table 1). The higher fructose consumption of E1 was not directly related to mannitol production, and only E1 strains produced mannitol, however, both strains produced erythritol, and E1 produced the highest concentration of this metabolite when compared to PSU-1 (Table 1).

#### White wine variety Chardonnay (pH 3.2)

Similarly, E1 in white wine consumed a higher total of fructose, citrate, and L-malate, when compared to PSU-1; and it produced higher (D + L) lactate concentration (Table 1). However, both strains produced a similar total erythritol concentration, and neither strain showed mannitol production.

In relation to the total acetate produced, E1 strain produced a higher concentration than the PSU-1 strain in both wines. In Chardonnay, both strains produced much lower acetate concentration than in MaxOeno (28 times less in PSU-1 and at least 23 time less in E1) (Table 1). However, it is important to highlight that PSU-1 produced less biomass (62% less) and E1 produces more biomass in Chardonnay (46% more) compared to its production in MaxOeno (Table 1).

#### Effect of wine type on the percentages of conversion of malate of *Oenococcus oeni* PSU-1 and E1 strain

Regarding the percentages of conversion of malate, they were calculated in the different culture conditions for both strains. In all cases, it was observed that the E1 mutant strain was able to convert a higher L-malate percentage in comparison to PSU-1, showing significant statistical differences (Table 2). Thereby, the E1 strain was able to convert an average of 64% more of L-malate in comparison to PSU-1, regardless of the wine being used.

**Table 2:** Malate conversion generated by PSU-1 and E1 strains in different culture conditions (*).

Wine	Concentration of malate residual (£) (g L^−1^)		Malate conversion (%)	
	PSU-1	E1	PSU-1	E1
MaxOeno pH 3.5	5.98^Ba^ ± 0.23	5.60^Bb^ ± 0.03	13.51^Ba^	19.69^Bb^
Cabernet Sauvignon	0.77^Ca^ ± 0.01	0.01^Cb^ ± 0.01	64.82^Ca^	99.72^Ca^
Chardonnay	0.99^Ca^ ± 0.31	0.46^Db^ ± 0.07	46.16^Aa^	73.25^Db^

(*) Same letters in the same row or column indicate that there are no statistical significant differences (*p* < 0.05, Mood test) between the reported values. Likewise, capital letters indicate differences or similarities between treatments and lower cases indicate differences or similarities between strains. (£) The concentration of malate at the end of MLF. The initial malate concentration in MaxOeno pH 3.5, Cabernet Sauvignon and Chardonnay were 6.9 ± 0.2, 2.2 ± 0.2, 1.9 ± 0.1, respectively.

### Effect of the type of wine on the production of metabolites and consumption of substrate of *Oenococcus oeni* PSU-1 and strain E1

#### Non-growth associated maintenance

Flux balance analysis was carried out using the metabolic model iSM454, developed by Mendoza et al. (2017), to analyze the highest growth phase of the PSU-1 and the E1 strain in the different culture media used. Even though the PSU-1 strain cultivated in Chardonnay wine did not have considerable growth, the flux redistribution analysis was carried out to have an approximate vision of what happens with the metabolism of this strain. Prediction of Non-growth Associated to Maintenance (NGAM) was assessed for synthetic (MaxOeno), red (Cabernet Sauvignon), and white wine (Chardonnay). In synthetic wine, NGAM was 9.54 and 5.48 (mmol gDCW^−1^ h^−1^) for PSU-1 and E1, respectively. In red wine, NGAM was 0.30 and 0.64 (mmol gDCW^−1^ h^−1^) for PSU-1 and E1, respectively. Finally in white wine, it was 1.36 and 0.74 (mmol gDCW^−1^ h^−1^) for PSU-1 and E1, respectively. In general, it was observed that both strains had a NGAM higher in MaxOeno (average 90% more) compared to natural wines. The E1 strains showed lower NGAM in MaxOeno and Chardonnay (average 42% less) than PSU-1 strain; however, the E1 strain showed higher NGAM (50% more) in Cabernet Sauvignon compared to PSU-1 strain.

### Redistribution of metabolic fluxes

**Figure 3:**
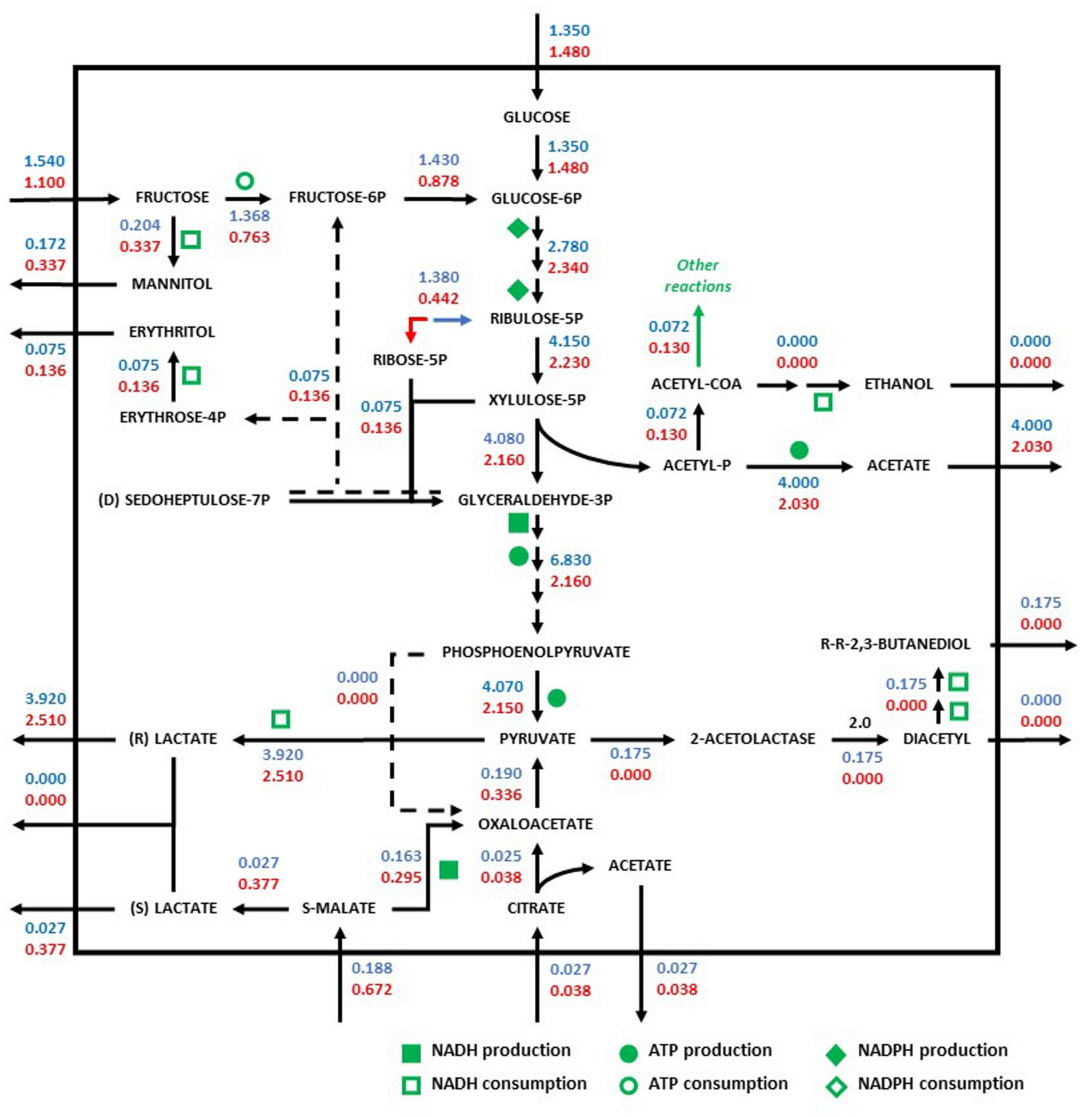
Redistribution of the metabolic flux of the strains *Oenococcus oeni* PSU-1 (blue) and *O. oeni* E1 (red) evaluated using MaxOeno. Square, circle, and diamond polygons indicate consumption (or production) of NADH, ATP, and NADPH, respectively. S-malate corresponds to L-malate. S-and R- lactate correspond to L-and D lactate, respectively.

**Figure 4:**
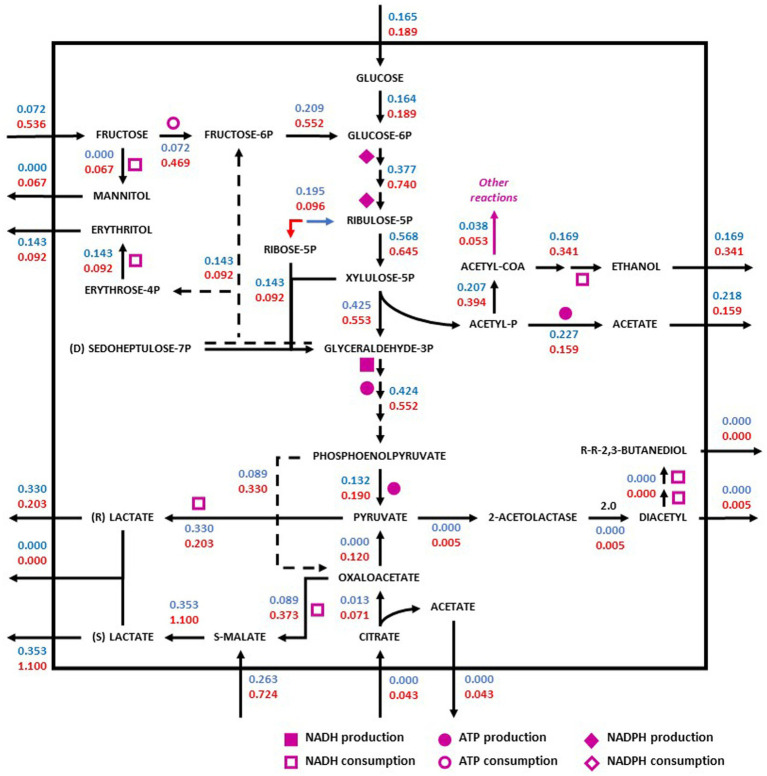
Redistribution of the metabolic flux of the strains *O. oeni* PSU-1 (blue) and *O. oeni* E1 (red) evaluated using Cabernet Sauvignon. Square, circle, and diamond polygons indicate consumption (or production) of NADH, ATP, and NADPH, respectively. S-malate corresponds to L-malate. S-and R- lactate correspond to L-and D lactate, respectively.

**Figure 5:**
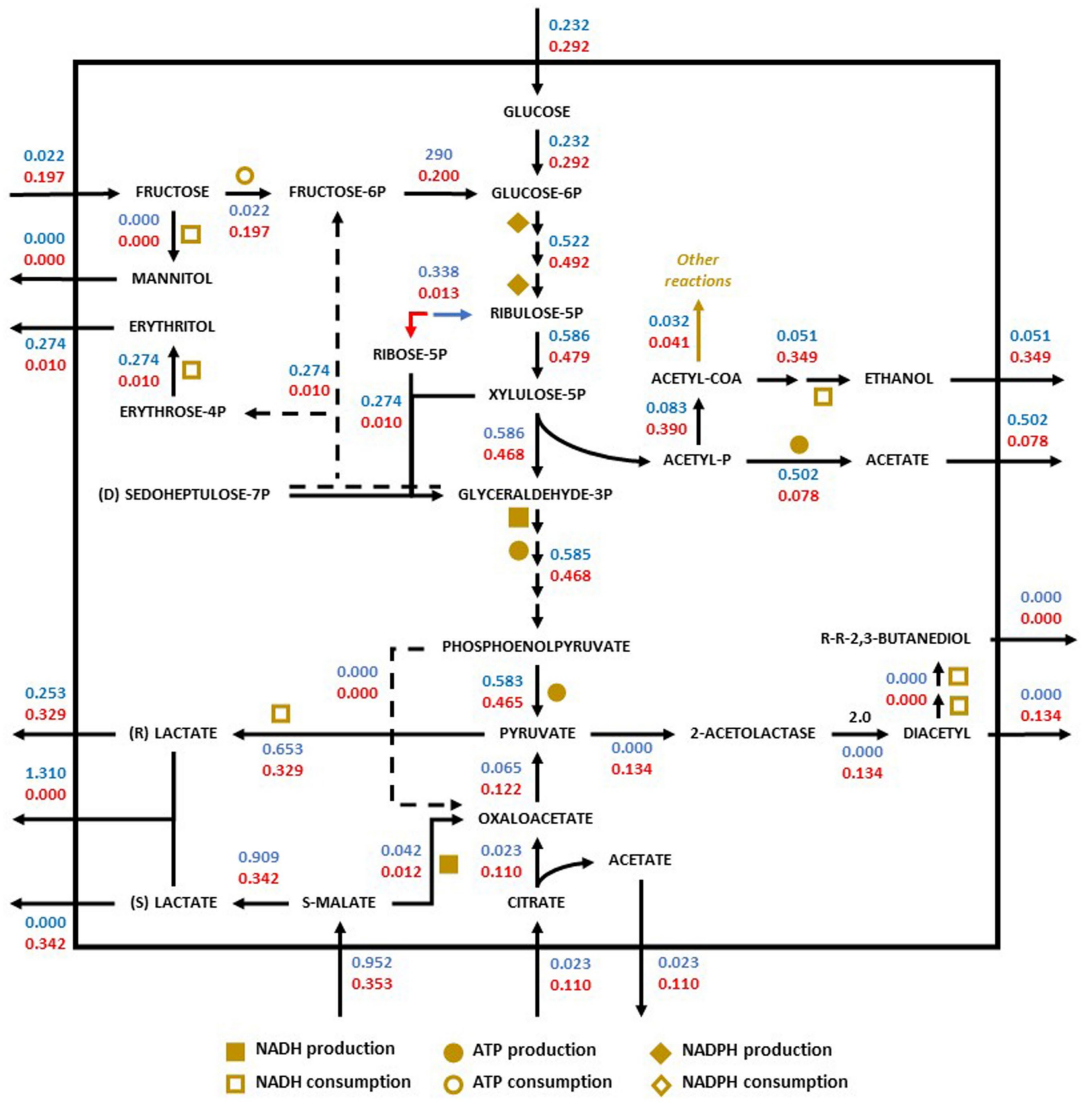
Redistribution of the metabolic flux of the strains *O. oeni* PSU-1 (blue) and *O. oeni* E1 (red) evaluated using Chardonnay. Square, circle, and diamond polygons indicate consumption (or production) of NADH, ATP, and NADPH, respectively. S-malate corresponds to L-malate. S-and R- lactate correspond to L-and D lactate, respectively.

Redistribution of the metabolic flux of the strains *O. oeni* PSU1 (blue) and *O. oeni* E1 (red) was evaluated in three wine culture conditions (Figures 3–5). The metabolic pathways of carbon were studied using the iSM454 model and the pathway of phosphoketolase (heterolactic fermentation), reduction of fructose, and degradation of citrate, and malolactic fermentation showed differences between the strains evaluated (Figures 3–5).

In synthetic wine (MaxOeno), the PSU-1 strain showed a higher specific rate of fructose consumption (1.54 mmol gDCW^−1^ h^−1^) when compared to the E1 strain (1.10 mmol gDCW^−1^ h^−1^) (Figure 2). However, the E1 strain showed a higher specific rate of mannitol production (0.34 mmol gDCW^−1^ h^−1^) than PSU-1 (0.17 mmol gDCW^−1^ h^−1^). Both strains showed similar specific rates of glucose consumption. Finally, the E1 strain showed a higher consumption of L-malate (0.67 mmol gDCW^−1^ h^−1^), and a higher (D + L)-lactate production (0.38 mmol gDCW^−1^ h^−1^), comparted to PSU-1 (0.19 mmol gDCW^−1^ h^−1^ and 0.03 mmol gDCW^−1^ h^−1^, respectively) (Figure 3).

In red wine (Cabernet Sauvignon), on one hand, the E1 strain showed a higher specific rate of fructose consumption (0.54 mmol gDCW^−1^ h^−1^) compared to the PSU-1 strain (0.07 mmol gDCW^−1^ h^−1^) (Figure 4), showing a high specific rate of mannitol production (0.07 mmol gDCW^−1^ h^−1^) as well. Moreover, the E1 strain showed a higher consumption of L-malate (0.72 mmol gDCW^−1^ h^−1^), and a higher (D + L)-lactate production (1.10 mmol gDCW^−1^ h^−1^), comparted to PSU-1 (0.26 mmol gDCW^−1^ h^−1^ and 0.35 mmol gDCW^−1^ h^−1^, respectively) (Figure 4). On the other hand, the PSU-1 strain showed a higher specific rate of erythritol (0.14 mmol gDCW^−1^ h^−1^) and D + L-Lactate production (0.33 mmol gDCW^−1^ h^−1^) compared to the E1 strain (0.09 mmol gDCW^−1^ h^−1^ and 0.20 mmol gDCW^−1^ h^−1^, respectively) (Figure 4). Finally, E1 strains showed a low specific rate of the heterolactic pathway of acetate production (0.08 mmol gDCW^−1^ h^−1^), and a higher specific rate of ethanol production (0.34 mmol gDCW^−1^ h^−1^), compared to PSU-1 strain (0.22 mmol gDCW^−1^ h^−1^ and 0.17 mmol gDCW^−1^ h^−1^, respectively).

In white wine (Chardonnay), the E1 strain preferentially consumed L-malate (0.35 mmol gDCW^−1^ h^−1^) and L-citrate (0.11 mmol gDCW^−1^ h^−1^), producing oxaloacetate and then pyruvate (0.12 mmol gDCW^−1^ h^−1^). In contrast, PSU-1 consumed lower nutrient compared to E1 (Figure 5).

In general, it was observed that only E1 produces mannitol and diacetyl; PSU-1 does not produce them and only produces mannitol in red wine, and diacetyl only in white wine. We observed that the consumption of L-malate for the production of L-lactate was 82%, and the consumption of fructose and glucose to generate pyruvate and D-lactate, through its heterolactic route, was 18%.

### Identification of genes that showed differences between the mutant strain and the parental strain at the transcriptomic level using MaxOeno with a 15% v/v of ethanol at pH 3.5

We performed a massive transcriptome sequencing to identify changes produced by random mutagenesis. Then, eight genes of known function were observed that showed differences between the mutant strain and the parental strain, and from these genes, four were selected to be analyzed by qPCR. According to their description, these genes would be related to growth and fructose consumption, which were the highest differences observed between E1 and PSU-1 during the culture analyses presented above. The OEOE_1708 gene codes for a fructokinase enzyme (EC 2.7.1.4) involved in the transformation of fructose to fructose-6-phosphate. This overexpression was determined approximately at 16 h of culture and coincides with the greatest difference between the strains, considering the consumption of fructose and the production of acetate (Supplementary Figures S5B,D, Supplementary material). These results were validated using the quantitative chain reaction (qPCR) technique in order to confirm the highest expression observed (Table 3). The OEOE_1794 (codes a universal stress protein) and OEOE_1795 (codes a Mn^2+^ and Fe^2+^ transporter) genes showed a higher number of transcripts in E1 than in PSU-1 when both transcriptomic analysis techniques were used. However, the OEOE_0522 gene (codes a transcriptional regulator, PadR family) showed a higher number of transcripts in E1 than in PSU-1 when using the HiSeq sequencing technique but it was not satisfactorily amplified when qPCR was used.

**Table 3:** Gene expression rate of E1 compared to PSU-1 (*).

Open reading frame	Relative gene expression QPCR	Relative gene expression ratio (¥)	Gene expression fold change (¥)	Description (**)
OEOE_1794	14.4	9.8	3.3	Universal stress protein UspA-like
OEOE_1795	2.0	8.5	3.1	Mn^2+^ and Fe^2+^ transporter of the NRAMP family
OEOE_1708	6.9	5.2	2.4	Fructokinase
OEOE_0522	NA	4.9	2.3	Transcriptional regulator, PadR family

(*) transcripts observed in E1 compared to PSU1. (**) NCBI and KEGG database and bibliography were used to make the description of these genes. (¥) Massive sequencing. NA it not amplified.

## Discussion

### Evaluation of the mutant strain E1 and its parental strain PSU-1, using MaxOeno at pH 4.8, as the last selection step

This study analyzed the capacity of the random mutagenesis technique using EMS as a strategy for the genetic improvement of strains of the *Oenococcus oeni* species, which, to our knowledge, has not been applied to this species before. As the last selection step, the mutagenized strain E1 and the parental strain PSU-1 were cultivated in synthetic wine (MaxOeno) at pH 4.8, and then their metabolite consumption and production capacities were compared. Based on the results, this technique proved able to generate a new and improved strain in comparison to its parental PSU-1 strain, from which it descends. It is important to point out that the growth and metabolization traits of the malate from the E1 strain were maintained by the strain through every trial performed in this work, without modifying, its behavior which indicates that the stress and selection conditions to which the cell was subjected to generated persistent modifications that benefit its survival. Some authors who have used different techniques for natural genetic improvement (without using genetic engineering) have observed that applying stress and selection rounds are key processes for maintaining a phenotype (Akcil, 2004; Li et al., 2015; Betteridge et al., 2018; Jiang et al., 2018).

### Effect of wine type on the growth of *Oenococcus oeni* PSU-1 and E1 strain

To evaluate the oenological capacities of both strains, and identify their differences, we analyzed their metabolic behavior in three different wines, using the iSM456 metabolic model constructed for *O. oeni*. Even more, we identified genes that showed differences in expression when comparing both strains.

In our previous work, we observed that the specific growth rate linearly decreases when the ethanol content in the wine increases (Contreras et al., 2018). We observed similar trends in the present results with the PSU-1 strain cultivated in MaxOeno with 15% of ethanol (r2 = 0.96). Conversely, the mutant strain E1, in average, showed a specific growth rate that was 39% greater than PSU-1 in synthetic wine and red wine. In wine white, the PSU-1 strain showed little growth, hence, E1 strain showed a specific growth rate that was 86% greater than PSU-1. Moreover, the E1 strain was able to convert an average of 34% more of malate in comparison to PSU-1, regardless of the wine being used.

PSU-1 strain showed a greater production of total biomass in red wine (Cabernet Sauvignon), although it was less in comparison to the E1 strain, and a minimal production of biomass in white wine. It has been noted that strains that have evolved in a particular environment develop the ability to adapt and take advantage of nutritional and physical–chemical conditions (Campbell-Sills et al., 2015; Breniaux et al., 2018; Collombel et al., 2019). Although all wines contain phenolic compounds, red wines are the ones which have the highest concentration of it (Paixão et al., 2007). Among the red wines in Chile, the Cabernet Sauvignon variety has shown the highest concentration of phenolic compounds (Lutz et al., 2012). Likewise, it has been stated that phenolic compounds aid in the survival of microorganisms since they function as controlling agents of the redox potential (Rozès et al., 2003). The PSU-1 strain of *O. oeni* was isolated from red wine in Pennsylvania, in 1972, and one of its main advantages has been described as its capacity to induce MLF faster in red wines (Beelman et al., 1977). It is possible that the PSU-1 strain evolved in red wine grapes and that this is why phenolic compounds might help its metabolism and resistance to stress.

### Effect of wine type on metabolites production and substrate consumption of *Oenococcus oeni* PSU-1 and E1 strain

Other than cellular growth, among the main differences observed between E1 and PSU-1 was the differentiated intake of nutrients. Regardless of the type of wine being used, the mutant E1 strain showed a higher total consumption of fructose, malate, and citrate, which is also related to a high production of mannitol and lactate. Some authors state that the concentration of citrate and/or malte in the culture media could trigger a change in the preference of nutrient consumption (Ramos and Santos, 1996; Rozès et al., 2003). However, we observed that its consumption was mainly promoted by metabolic needs the bacteria generates to survive stress. This was observed in the results of consumption and production of compounds expressed in g/L, and, also, in the results of specific rates expressed in mmol gDCW^−1^ h^−1^.

### Data integration using the genomic scale metabolic iSM454 model

#### Non-growth associated maintenance

The energy needed by these organisms is composed by growth associated maintenance energy (GAM) and non-growth associated maintenance (NGAM) (Pirt, 1965; Neidhardt et al., 1992; Ulas et al., 2012; Hoehler and Jørgensen, 2013). The cost of GAM represents the necessary energy (ATP) for mechanisms like replication, transcription, and translation to produce biomass. NGAM represents the cell’s requirements to maintain biological functions that allow its survival such as its membrane potential, protein folding, or DNA repair (Ulas et al., 2012; Hoehler and Jørgensen, 2013).

In our previous work, the PSU-1 strain showed a 30-fold higher flux rate, in the reactions that produce maintenance energy (NGAM), when grown in MaxOeno with 12% ethanol as opposed to 0% ethanol (Mendoza et al., 2017). It is observed that as the ethanol concentration increased from 0 to 12%, the NGAM value increased, so we concluded that this increase was due to the stress generated by the presence of ethanol (Contreras et al., 2018). In the present work, both strains presented high values of NGAM because both strains were subjected to the same stress by ethanol and low pH. Noteworthy, it was observed that PSU-1 cultured in MaxOeno and Chardonnay showed an average of 42% more NGAM flow rate compared to E1. This suggests that E1 could be more resistant to the stress present in the wine.

Incidentally, it was also observed, in Cabernet Sauvignon red wine, that the specific fluxes in the reactions that produce maintenance energy (NGAM) was greater (50% more) in the E1 strain than in the PSU-1 strain. Moreover, in red wine both strains had lower flow rate in total NGAM in comparison to the other wines. The PSU-1 strains had a total NGAM of 0.3 mmol gDCW^−1^ h^−1^, similar to the values obtained under the stress of 3% (Contreras et al., 2018). In contrast, the E1 strain cultured in red wine showed a total NGAM value of 0.6 mmol gDCW^−1^ h^−1^, similar to the values obtained under the stress of 6% ethanol (Contreras et al., 2018).

### Redistribution of metabolic fluxes

We observed that the glucose consumed preferably follows the heterolactic route for the production of pyruvate. What is more, most of the consumed fructose by both strains goes into the production of pyruvate instead of mannitol. In our previous work, we observed that *O. oeni* generated NAD(P)^+^ cofactors through the production of mannitol and erythritol, contributing to the balance of internal pH through the production of diacetyl; results that were also observed by other authors (Saguir and De Manca Nadra, 1996; Versari et al., 1999).

Likewise, when analyzing the intracellular specific fluxes of E1, it was observed that the production of fructose-6-phosphate, which impacts the pyruvate production route, was 86% higher than the production rate of mannitol. In addition, in this strain, the consumption rate of malate is 26 and 74% higher than its consumption rate of fructose and glucose, respectively.

Mannitol production is limited and, thereby, NAD(P)^+^ production is as well. This might be due to the lack of manganese (Mn^2+^) in the cell; this element is key for malolactic enzyme activation (Acevedo et al., 2020) and for the function of numerous enzymes related to pyruvate production (Saha, 2006). Moreover, it has been observed that a low Mn^2+^ concentration inside the cell inhibits mannitol production, even when the mannitol dehydrogenase enzyme does not require cofactors to function (von Weymarn et al., 2002). Authors who have studied the proteome and the transcriptome of bacteria have observed the presence of Mn^2+^ and the response regulation to varied types of stressors, and even an impact on the physiology and metabolism of these microorganisms (Ogunniyi et al., 2010; Mbah and Isokpehi, 2013).

### Identification of genes that showed differences between the mutant strain and the parental strain

Interestingly, the E1 strain showed an overexpression of the OEOE_1794 gene, which codes an UspA-like protein which allows for cell survival and growth under stress conditions. The family of universal stress proteins (UspA-like) has been described as one of the main tools for allowing bacteria, archaea, fungi, and plants to be able to respond to unfavorable stress conditions so that they adapt, survive, and grow (Nachin et al., 2005; Isokpehi et al., 2011; Mbah and Isokpehi, 2013). The deletion of USP genes affect the growth of many bacteria such as *Escherichia coli*, *Mycobacterium tuberculosis*, *Salmonella typhimurium*, *Burkholderia pseudomallei,* and *Listeria monocytogenes* (Chatterjee et al., 2006; Liu et al., 2007; Hingley-Wilson et al., 2010; Al-Maleki et al., 2014).

This concurs with the higher number of transcripts from the OEOE_1708 gene observed in the E1 strain grown in MaxOeno. This gene codes for a fructokinase enzyme (EC 2.7.1.4) involved in the transformation of fructose into fructose-6-phosphate. This is consistent with findings described by other authors who have observed that pyruvate formation is the cell’s key strategy to maintain pH homeostasis in low pH conditions, meaning in acid stress (Fernandez et al., 2008; Zuljan et al., 2014; Qi et al., 2021). In our case, stress is increased by the presence of high concentrations of ethanol, which generates fluidization in the plasmatic membrane and the influx of ions (Da Silveira et al., 2002; Chu-Ky et al., 2005). The above would be explained because pyruvate is a key compound that binds the metabolism of carbon to other metabolic routes related to the production of amino acids and fatty acids, mainly (Liu et al., 2007; Maleki and Eiteman, 2017; Suo et al., 2021). This indicates that the cell could be activating this route to produce systems to repair the membrane like the production of fatty acids and ATP production. It has been observed that the [NAD(P)H/NAD(P)+] redox balance inside the cell has direct impact on the production rate of pyruvate. Some authors who have worked with *Escherichia coli* have observed that NAD(P) + increases glucose consumption and pyruvate production (Hansen and Henning, 1966; Akita et al., 2016).

We observed that a greater expression of the OEOE_1795 gene codes for a protein that transports Mn^2+^ and Fe^2+^ (Mn^2+^ and Fe^2+^ transporter of the NRAMP family). This would indicate that the E1 strain is resistant to low pH and high ethanol stresses when compared to the PSU-1 strain because it is more efficient in metabolizing malate, mannitol, and pyruvate as a result of its capacity to obtain Mn^2+^.

## Conclusion

In this study, we observed that it is possible to use random chemical mutagenesis on *O. oeni* to obtain improved strains there are able to withstand stressful environments like a high ethanol concentration in wine. We discovered that under the stress conditions of different wines the mutant strain E1 was able to grow and perform MLF more efficiently than its parental strain. Among the main strategies used by E1 used are a higher fructose and glucose consumption, and L-malate consumption for the production of L-lactate. We also observed that the consumption of fructose and glucose was related to a higher production of pyruvate, which can be used in several metabolic pathways, including fatty acid synthesis pathways. Moreover, the consumption of L-malate for the production of L-lactate is related to the redox balance of the cell, and we observed that genes related to the consumption of manganese in E1 favor this process. Finally, we observed a relation between higher growth showed by the E1 strain, in all of the wines evaluated, and the high relative expression of a gene related to UspA-like proteins synthesis, which allows survival and cellular growth.

It is necessary to carry on studying these genes, and the routes that metabolize the related compounds, so that their biological function in *O. oeni* and its impact in MLF can be verified.

## Data availability statement

The datasets presented in this study can be found in online repositories. The names of the repository/repositories and accession number(s) can be found at: https://www.ncbi.nlm.nih.gov/, GSE217343 (token mberokakzfirnyl) and https://github.com/Maucicio/Strain-analysis-main-Public-.

## Author contributions

EA designed the research and provided guidance throughout the investigation. ÁC wrote the paper, designed, performed, and coordinated the batch experiments; transcriptomic analysis and mutagenesis process, as well as processed the HPLC data. GD contributed to the evaluation of the strains obtained in the different types of. MC executed the modeling simulations and analyses. SM supervised these analyses. All the authors read, corrected, and approved the final manuscript.

## Funding

The work was supported by the FONDECYT Postdoctoral Research Fellowship N°3150151 supported ÁC.

## Conflict of interest

The authors declare that the research was conducted in the absence of any commercial or financial relationships that could be construed as a potential conflict of interest.

## Publisher’s note

All claims expressed in this article are solely those of the authors and do not necessarily represent those of their affiliated organizations, or those of the publisher, the editors and the reviewers. Any product that may be evaluated in this article, or claim that may be made by its manufacturer, is not guaranteed or endorsed by the publisher.

## Footnotes

^1^ https://www.ncbi.nlm.nih.gov/geo/query/acc.cgi?acc=GSE217343

^2^ https://github.com/Maucicio/Strain-analysis-main-Public-
