# Supplementary material for: Metabolic behavior for a mutant Oenococcus oeni strain with high resistance to ethanol to survive under oenological multi-stress conditions
Source: Front Microbiol. 2023 Mar 9;14:1100501. doi: 10.3389/fmicb.2023.1100501 (PMC10033693; doi:10.3389/fmicb.2023.1100501)
Supplement: Supplementary file 1 [file Data_Sheet_1.PDF]

Supplementary material

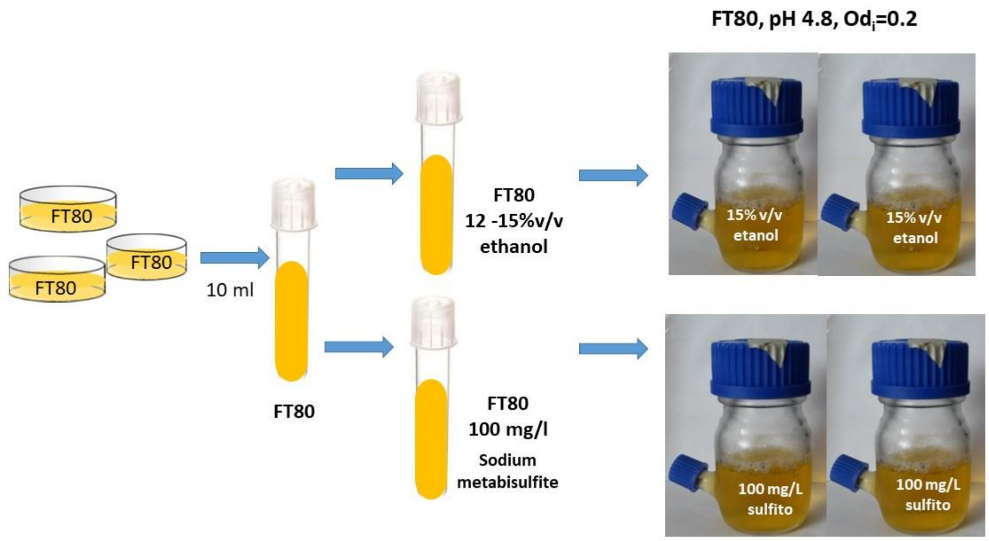

Figure S1. Mutant selection process scheme

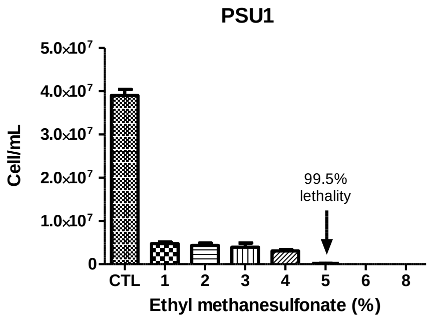

Figure S2. Lethality curve for Ethylmethane sulfonate utilization on *O. oeni* PSU-1 strain.

Table S1. Initial test of resistance to ethanol and potassium metabisulfite of O. oeni PSU1strain.

	**Crecimiento (¥)**
**PSU1 10% v/v ethanol**	+
**PSU1 15% v/v ethanol**	-
**PSU1 20% v/v ethanol**	-
**PSU1 50 mg/L metabisulfite**	+
**PSU1 100 mg/L metabisulfite**	-
**PSU1 200 mg/L metabisulfite**	-

¥ Growth observed to 9 days of culture

Table S2. Oligonucleotides used in this study

Genes	Primer sequences (5´–3´)
OEOE_1794	GGTGAAAGCCGATCTCTTGA
	AAATTGGCGCATATTTAGCC
OEOE_1795	ATGCGAATGGTCCTTCTTTG
	ATCCAACCGCTACAAGTGCT
OEOE_1708	TTGAAAAAGGTTCGTGTCCA
	AAATCGCCTAAAGTCGCTGA
OEOE_0522	TCCCCAAGGGAACGATTTAT
	TTTCTCCCAAACCTTTTTCG
OEOE_0005	GAGGATGTCCGAGAAGGAATTA
**reference**	ACCTGCTGGGCATCTGTATTG

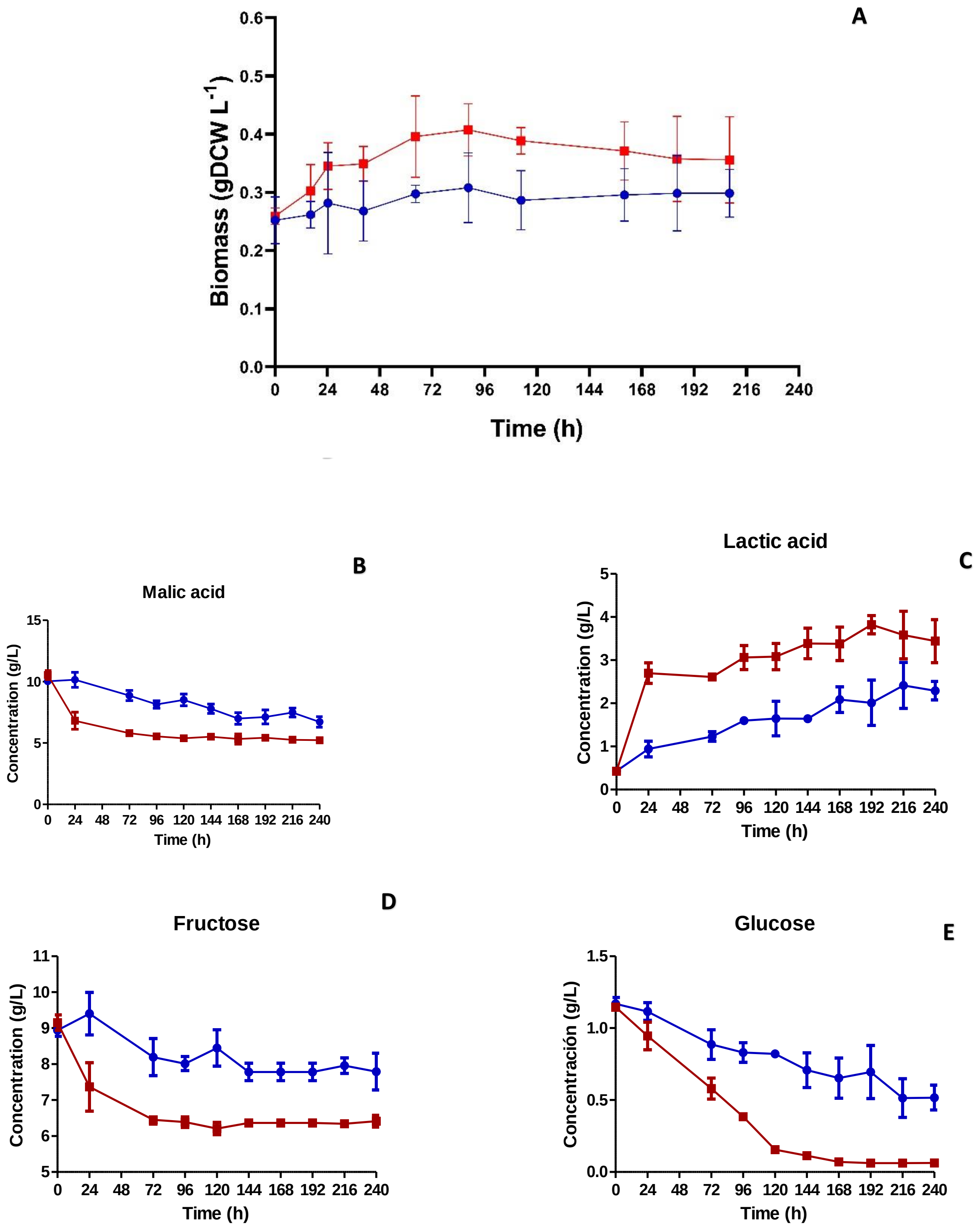
A
Biomass (gDCW L-1)
0.0
0.1
0.2
0.3
0.4
0.5
0.6
0 24 48 72 96 120 144 168 192 216 240
Time (h)
B
Malic acid
Concentration (g/L)
0 5 10 15
0 24 48 72 96 120 144 168 192 216 240
Time (h)
Lactic acid
C
Concentration (g/L)
0 1 2 3 4 5
0 24 48 72 96 120 144 168 192 216 240
Time (h)
D
Fructose
Concentration (g/L)
5 6 7 8 9 10 11
0 24 48 72 96 120 144 168 192 216 240
Time (h)
Glucose
E
Concentración (g/L)
0.0 0.5 1.0 1.5
0 24 48 72 96 120 144 168 192 216 240
Time (h)

Figure S5. Kinetics of production and consumption compounds of the E1 (red) and PSU1 (blue) strains, grown in MaxOeno with 15% ethanol at pH 4.8. Biomass production (A), Malic acid consumption (B), and lactic acid production (C). Fructose (D) and glucose (E) consumption.

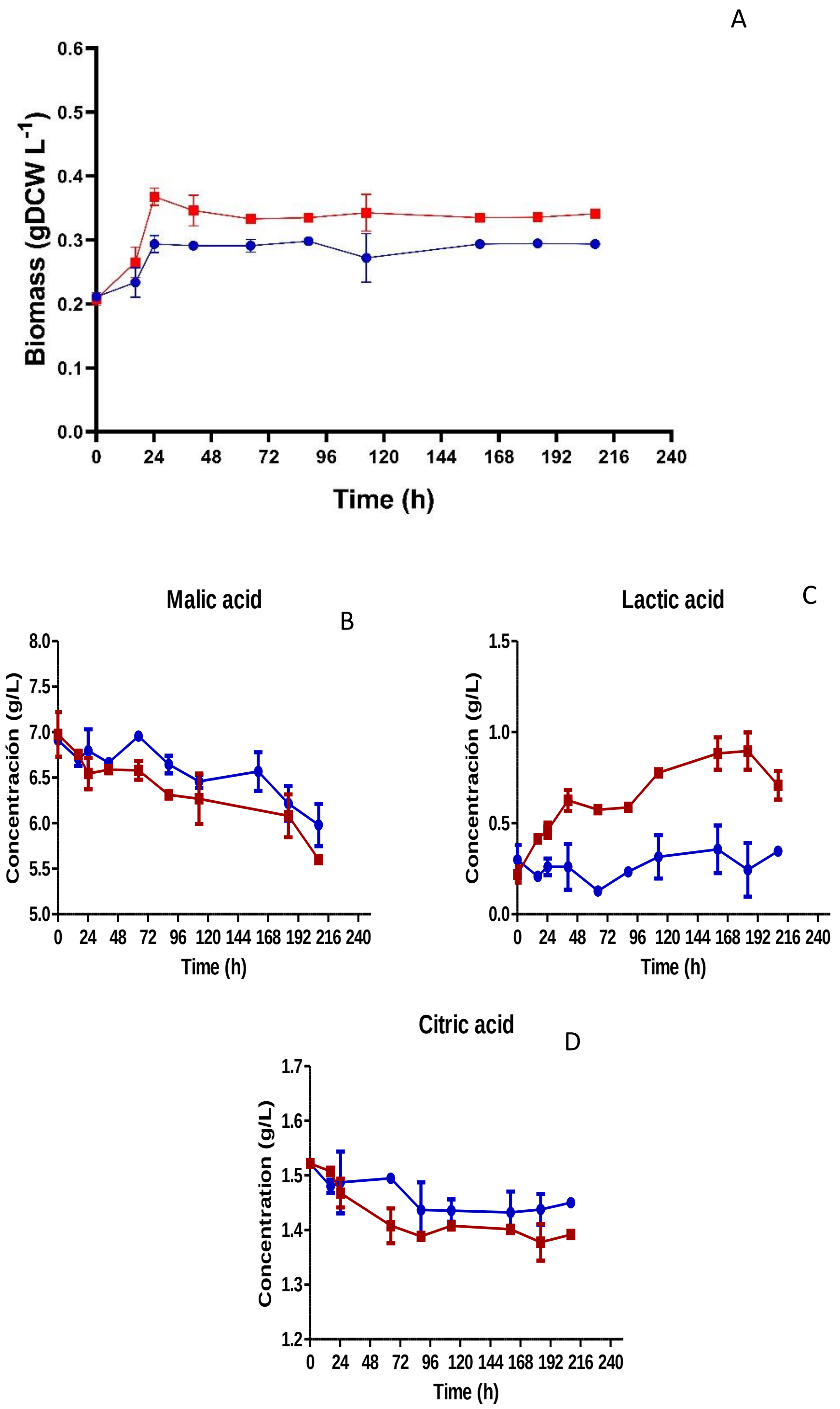

Figure S6. Kinetics of production and consumption compounds of the E1 (red) and PSU1 (blue) strains, grown in MaxOeno with 15% ethanol at pH 3.5. Biomass production (A), Malic acid consumption (B), lactic acid production (C) and citric acid consumption (D).

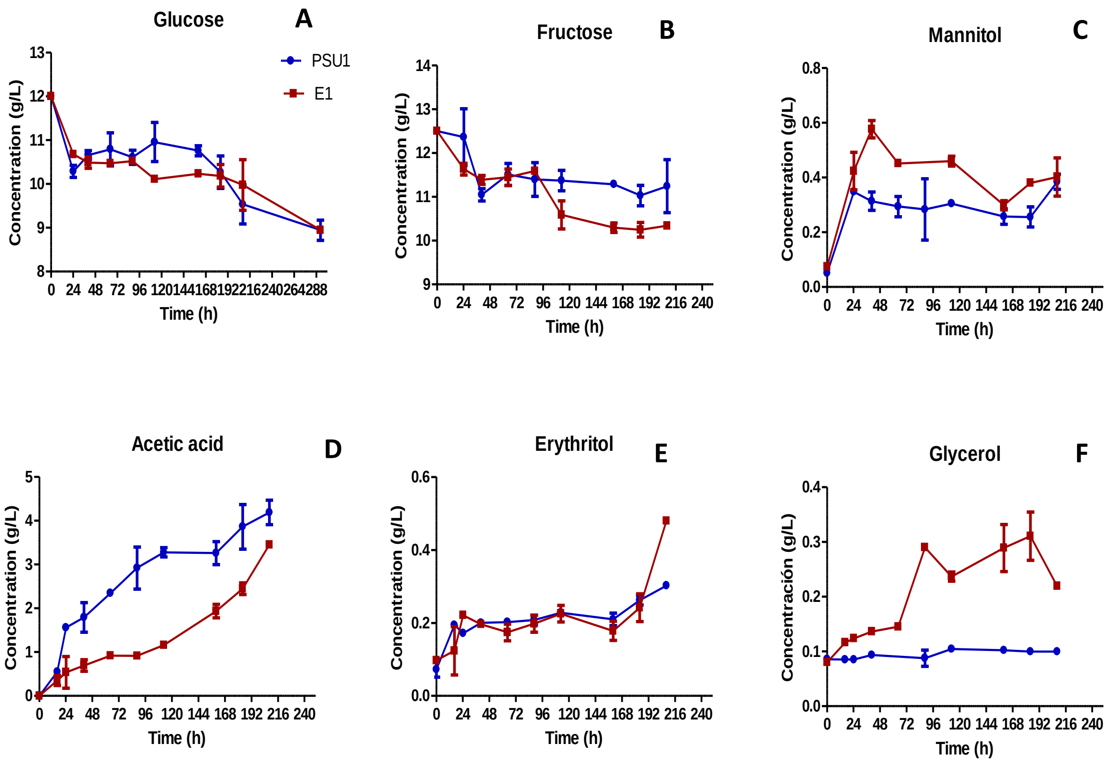

Figure S7. Kinetics of production and consumption compounds of the E1 (red) and PSU1 (blue) strains, grown in MaxOeno with 15% ethanol at pH 3.5. Glucose consumption(A), Fructose consumption(B), Mannitol production(C), Acetic acid production (D), Erythritol production (E), and Glycerol production (F).
